# Interleukin-1 Beta Gene Polymorphism rs16944 May Associate with Increased Susceptibility to Extremity Chronic Osteomyelitis in Chinese Han Population

**DOI:** 10.1155/2019/7483537

**Published:** 2019-03-04

**Authors:** Zi-long Yao, Qing-rong Lin, Yan-jun Hu, Yi-long Hou, Yun-fei Ma, Bin Yu, Nan Jiang

**Affiliations:** ^1^Department of Orthopaedics & Traumatology, Nanfang Hospital, Southern Medical University, Guangzhou 510515, China; ^2^Guangdong Provincial Key Laboratory of Bone and Cartilage Regenerative Medicine, Nanfang Hospital, Southern Medical University, Guangzhou 510515, China

## Abstract

**Background:**

Previous studies had indicated that interleukin-1 beta (IL-1*β*) gene single nucleotide polymorphisms (SNPs) associate with different inflammatory diseases. However, potential links between these polymorphisms and susceptibility to extremity chronic osteomyelitis (COM) remain unclear. This study aimed to investigate relationships between IL-1*β* gene polymorphisms (rs16944, rs1143627, rs1143634, and rs2853550) and risks of developing extremity COM in Chinese Han population.

**Methods:**

Altogether 233 extremity COM patients and 200 healthy controls were genotyped for the four tag SNPs of the IL-1*β* gene using the SNapShot genotyping method. Comparisons were performed regarding genotype distribution, mutant allele frequency, and four genetic models (dominant, recessive, homozygous, and heterozygous models) of the four SNPs between the two groups.

**Results:**

Significant associations were identified between rs16944 polymorphism and the risk of developing COM by dominant model (*P* = 0.026, OR = 1.698, 95% CI 1.065-2.707) and heterozygous model (*P* = 0.030, OR = 1.733, 95% CI 1.055-2.847). Although no statistical differences were found of rs1143627 polymorphism between the two groups, there existed a trend that rs1143627 may be linked to an elevated risk of developing COM by outcomes of dominant (*P* = 0.061), homozygous (*P* = 0.080) and heterozygous (*P* = 0.095) models. However, no statistical correlations were found between rs1143634 and rs2853550 polymorphisms and susceptibility to COM in Chinese Han population.

**Conclusions:**

To our knowledge, we reported for the first time that IL-1*β* gene rs16944 polymorphism may contribute to the increased susceptibility to extremity COM in Chinese Han population, with genotype of AG as a risk factor.

## 1. Background

Extremity chronic osteomyelitis (COM), characterized by inflammatory associated bone destruction with concurrent of new bone formation, usually arises as a sequelae or complication following trauma or orthopaedic surgery [1]. Currently, COM remains to be one of the most challenging disorders in front of orthopaedists, owing to its long disease course, complex in treatment with a higher risk of infection recurrence [2]. In addition, COM also means a catastrophic consequence for patients. Firstly, COM patients have higher incidences of physical and psychological disabilities [3]. Secondly, recent studies have demonstrated that patients with COM might suffer from increased susceptibility to other accompanying diseases, such as rheumatoid arthritis [4], acute pancreatitis [5], diabetes mellitus [6], and intracerebral hemorrhage [7]. Thirdly, diagnosis, treatment, and rehabilitation of COM bring great socioeconomic burdens [8]. Therefore, in addition to clinical studies, great efforts should be taken to investigate pathogenesis of this disorder in order to better solve COM related problems.

Pathogenesis of COM is linked to both environmental and host factors. As an important aspect of host factors, genetic variations play a vital role in the occurrence and development of COM. Recently, growing evidence has revealed that several single nucleotide polymorphisms (SNPs) may be correlated with the risk of developing COM, such as vitamin D receptor (VDR) genes* TaqI *(rs731236) and* FokI *(rs2228570) [9],* cyclooxygenase-2* (COX-2) gene rs689466 [10],* tissue plasminogen activator* (*tPA*) gene* Alu* insertion/deletion (rs4646972) [11], and* matrix metalloprotease 1 *(*MMP-1*) gene rs1799750 [12].

It is known that interleukin-1 (IL-1) family cytokines involve in inflammation and immune-regulation and act as an important role in innate and adaptive immunity. Recent studies have indicated that IL-1 can directly affect bone homeostasis, and dysregulation of IL-1 has been proved to be associated with bone disorders [13]. IL-1 exists in two distinct forms, IL-1*α* and IL-1*β*, the latter of which has been reported to be involved in the pathogenesis of OM in recent animal studies [13, 14]. IL-1*β* cytokine is encoded by IL-1*β* gene, which is a highly polymorphic. After reviewing previously published studies, we found that four SNPs (rs16944, rs1143627, rs1143634, and rs2853550) located in IL-1*β* gene were the most frequently investigated in relation to the development of inflammatory diseases, such as pediatric Helicobacter pylori infection [15], congenital cytomegalovirus infection [16], septic shock [17], and seasonal influenza A/H3N2 virus infection [18].

Based on the recent outcomes regarding the fact that IL-1*β* participates in the pathogenesis of mice OM as well as the already established evidence that IL-1*β* polymorphisms are linked to inflammatory disorders, we hypothesized that IL-1*β* gene polymorphisms may be associated with COM development. Therefore, the current study aimed to investigate potential relationships between IL-1*β* gene polymorphisms (rs16944, rs1143627, rs1143634, and rs2853550) and the risks of developing COM in Chinese Han population.

## 2. Patients and Methods

### 2.1. Study Design, Population, and Setting

This study was designed as a case-control report. Participants were Chinese Han population with ethnicity of Asian. Eligible patients were those who sought medical attention for COM between August 2013 and October 2015 in Nanfang Hospital, Southern Medical University, a tertiary medical center in southern China. Diagnosis of COM was established based on any of the following three points: (1) histopathological examination of intraoperative specimen confirms infection, (2) a definite sinus or fistula connects directly to the bone or orthopaedic implant, and (3) cultures from at least two infection sites reveal the same pathogen. Eligible healthy controls, confirmed by physical examination center of our hospital, were those without any diseases or diseases histories. All the participants or legal guardian signed informed consent and the study was approved by medical ethics committee of the hospital.

### 2.2. SNP Genotyping

Ethylene diamine tetraacetic acid (EDTA) peripheral blood samples (2 ml) were collected for each participant. Four tag SNPs of the IL-1*β* gene (rs16944, rs1143627, rs1143634, and rs2853550) were genotyped using the Multiplex SNaPshot system (Applied Biosystems, Foster City, USA). The forward (F), reverse (R), and extension primers used for polymerase chain reaction (PCR) and extension reactions were listed in Table 1. Detailed study protocol was described previously [9].

### 2.3. Primary and Secondary Outcomes

Primary outcomes were comparisons regarding genotype distribution, mutant allele frequency, and four genetic models (dominant, recessive, homozygous, and heterozygous models) of the four IL-1*β* gene SNPs between patients and healthy controls. Secondary outcomes were comparisons of clinical features (age, sex ratio, positive rate of culture, percentage of polymicrobial infection, preoperative serum levels of white blood cell count (WBC), C-reactive protein (CRP), procalcitonin (PCT), interleukin-6 (IL-6), tumor necrosis factor-*α* (TNF-*α*), and serum amyloid A (SAA)) among different genotypes of the 4 IL-1*β* gene polymorphisms in the patient group.

### 2.4. Statistical Analysis

Statistical Product and Service Solutions version 13.0 (SPSS Inc., IL, USA) was used to conduct statistical analysis. Kolmogorov-Smirnov test was used to assess data distribution for normality. Continuous variables were presented as mean ± standard deviation or median with interquartile range (IQR) depending on data distribution. For normally distributed data, Student's t-test or one-way analysis of variance (ANOVA) was used to compare between 2 different groups or among over 2 groups. Otherwise, Mann-Whitney U test or Kruskal-Wallis H test was applied. Dichotomous variables were expressed as percentages and compared by Chi-square test or Fisher exact test.

Genotype distribution of healthy controls was tested for the confirmation to Hardy-Weinberg equilibrium (HWE) using the Chi-square test. The Chi-square test or Fisher exact test was used to compare genotype distributions and frequencies of mutant allele between the two groups. Binary logistic regression analysis with gender, age, and genotype distribution as covariates was used to evaluate potential associations between the four gene polymorphisms and the risk of developing COM by four genetic models, with corresponding odds ratios (ORs) and 95% confidence intervals (CIs). A* P* value of less than 0.05 was defined as statistical significance.

## 3. Results

### 3.1. Demographics

Altogether 233 patients (185 males and 48 females) with extremity COM and 200 healthy controls (147 males and 53 females) were included. The gender ratio between the two groups revealed no statistical difference (3.85 versus 2.77, *χ*^2^ = 2.094,* P* = 0.148). In addition, no significant difference was identified regarding the median age between the two groups, either (patient group: 42 years, IQR 28.50-54.00 versus control group: 41 years, IQR 36.25–47.00, Z = -0.169,* P* = 0.866).

### 3.2. Clinical Features of COM

COM clinical features of this Chinese cohort were described previously [9]. Posttraumatic OM was the most frequent type of COM (81.12%), which was primarily following open injury (66.30%). Tibia was the most common infection site (48.51%), followed by femur (29.70%), and toes and metatarsal bones (8.42%), respectively. Positive rate of intraoperative specimen culture was 64.74%, with monomicrobial infection accounted for 78.57%.* Staphylococcus aureus *(32.95%) lay in the top of the detected pathogen.

### 3.3. Frequency of the Four IL-1*β* Gene Polymorphisms in the Two Groups

All genotyped four IL-1*β* gene SNPs were in HWE for healthy controls (For rs16944, *P*_(HWE)_ = 0.161, for rs1143627, *P*_(HWE)_ = 0.399, for rs1143634, *P*_(HWE)_ = 0.829, and for rs2853550, *P*_(HWE)_ = 0.429).

As revealed in Table 2, significant associations were found between rs16944 SNP and susceptibility to COM by dominant model (*P* = 0.026, OR = 1.698, 95% CI 1.065-2.707) and heterozygous model (*P* = 0.030, OR = 1.733, 95% CI 1.055-2.847), suggesting that population with the genotype of AG may be a group in a higher risk to develop COM in Chinese Han population.

Although no statistical differences were found between rs1143627 and the risk of developing COM in this Chinese cohort, outcomes of the dominant (*P* = 0.061), homozygous (*P* = 0.080), and heterozygous (*P* = 0.095) models suggested a trend that population with TT and CT genotypes of this polymorphism may be groups in a higher risk to develop COM in Chinese Han population.

With respect to rs1143634 and rs2853550, no significant associations were observed between patients and healthy controls (Table 2).

### 3.4. Comparisons of Clinical Features among Different Genotypes of the IL-1*β* Gene Polymorphisms in COM Patients

**Figure 1:**
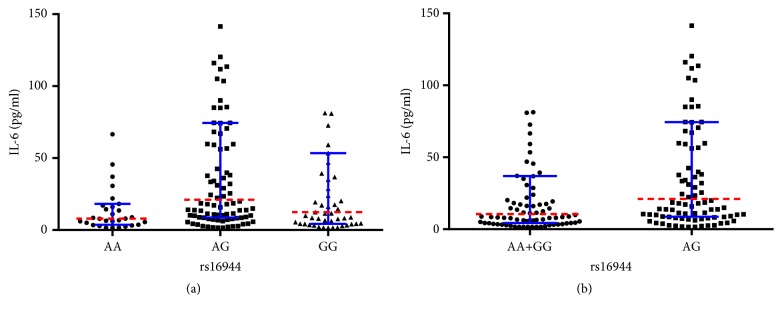
(a) Comparisons regarding preoperative serum IL-6 level among different genotypes of rs16944 in COM patients. (b) Comparisons regarding preoperative serum IL-6 level between AA+GG and AG groups in COM patients (red dotted line: median; blue error bars: IQR).

As shown in Table 3, significant differences were identified regarding preoperative serum levels of IL-6 among different genotypes of rs16944 (*P* = 0.005) and rs1143627 (*P* = 0.003). Outcomes of multiple comparisons regarding different genotypes of rs16944 among COM patients revealed that the median serum IL-6 level of AG genotype group was significantly higher than that of AA group (*P* = 0.001), with no statistical differences between AA versus GG (*P* = 0.164) or AG versus GG (*P* = 0.093) groups (Figure 1(a)). In addition, significant difference was found regarding median serum IL-6 level between AA+GG and AG genotype groups (10.51 pg/ml versus 21.10 pg/ml,* P* = 0.004) (Figure 1(b)).

**Figure 2:**
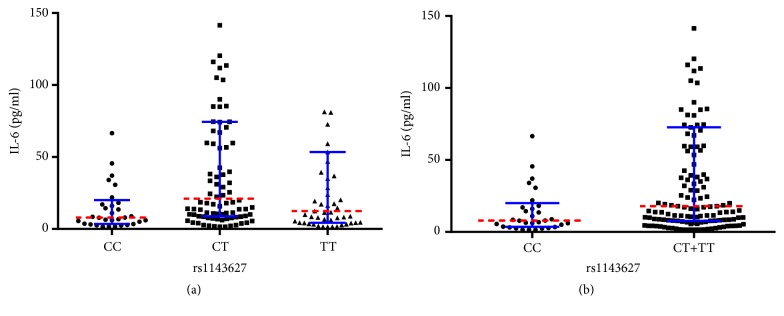
(a) Comparisons regarding preoperative serum IL-6 level among different genotypes of rs1143627 in COM patients. (b) Comparisons regarding preoperative serum IL-6 level between CC and CT+TT groups in COM patients (red dotted line: median; blue error bars: IQR).

Likewise, multiple comparisons regarding rs1143627 showed that the median serum IL-6 level of CT genotype group was statistically higher than that of CC group (*P* = 0.0008), with no significant differences between CC versus TT (*P* = 0.149) or CT versus TT (*P* = 0.079) genotype groups (Figure 2(a)). Additionally, significant difference was also observed regarding median serum IL-6 level between CC and CT+TT genotype groups (7.98 pg/ml versus 17.92 pg/ml,* P* = 0.004) (Figure 2(b)).

## 4. Discussion

Outcomes of the current study demonstrated that IL-1*β* gene rs16944 polymorphism may contribute to the elevated susceptibility to COM in Chinese Han population. People with AG genotype of rs16944 may be a group in a higher risk to develop COM. In addition, although the present study did not obtain statistical outcomes regarding rs1143627 polymorphism between patients and healthy controls, there existed a trend that rs1143627 polymorphism may correlate with an increased risk of COM development by outcomes of dominant, homozygous, and heterozygous models.

As mentioned previously, nowadays, COM, mostly arises following trauma or orthopaedic surgery, still represents great challenges and risks for clinicians. It was reported the incidence of infection following open fractures in the long bones ranged between 4% and 64%, with infection recurrence of 20% to even 30% [19]. Its long disease course and inflammatory bone destruction severely affect life qualities of the patients, both physically and psychologically. In a recent study, Huang et al. [20] indicated that COM significantly increased the risk of long-term mortality in the elderly. In addition to the previously mentioned comorbidities, deep venous thrombosis (VTE) [21], end-stage renal disease (ESRD) [22], dementia [23], erectile dysfunction [24] and even mental disorder like depression [3] also appear to be more frequent in COM patients. Moreover, there exists a risk of malignant transformation in patients with COM, though the incidence is low [25]. The primary disease and potential comorbidities increase economic burdens of the patients. A recent survey [8] revealed the treatment costs of infection after tibia fracture fixation were about 6.5 times higher than those without infection (€44, 468 versus € 6,855). Therefore, COM is not only a clinical disorder, but also a social problem.

It is known that occurrence of COM is influenced by external and internal factors. External factors include infection etiology (e.g., trauma, hematogenous spread, and diabetic foot), injury type and degree, situation of soft tissue, etc., whereas internal factors refers to host factors, such as age, smoking, alcoholism, nutrition situation, immune function, etc. [26]. Previous studies mostly focused on the effects of controllable external and internal factors on the incidence of COM and neglected uncontrollable genetic factors. Recently, increasing number of studies revealed potential roles of genetic factors in the pathogenesis of COM, with SNP as an important aspect. Up till now, several clinical investigations have reported significant links between gene SNPs and susceptibility to COM among different ethnicities, such as* VDR* gene [9],* COX-2 *gene [10],* tPA *gene [11],* MMP-1* gene [12], and the most frequently reported IL family genes.

Previous studies obtained positive outcomes of IL family gene polymorphisms associate with OM development, including IL-1*α* (rs1800587) [27, 28], IL-4 (rs2243248, rs2243250) [28], and IL-6 (rs1800795) [28]. All of the above gene polymorphisms increase the susceptibility to OM. As an important member of IL family cytokines, IL-1*β* has been identified to participate in the pathogenesis of OM in animal experiments [13, 14]. Considering the highly polymorphic of IL-1*β* gene as well as the confirmed relationships between IL-1*β* gene polymorphisms and the development of different inflammatory diseases, we hypothesized that IL-1*β* gene SNPs may be correlated with COM.

Outcomes of the current study demonstrated that IL-1*β* gene rs16944 may be related to the increased risk of developing COM in Chinese Han population. Similarly, in a recent study with 39 OM patients and 114 healthy controls, Alves De Souza et al. [29] found that rs16944 with rs2234663 polymorphisms may participate in the development of posttraumatic OM in Brazilian population. However, quite different from their findings of genotype of TT being more frequent in OM patients, we found AA was less while AG was more frequent in COM patients. Two possible reasons may account for the difference. One may lie in the different ethnicities. The other may attribute to the fact that in addition to posttraumatic OM, we also included COM patients resulting from hematogenous spread and diabetic foot. In another study consisting of 52 patients with hematogenous osteomyelitis (HO) and 103 controls, Osman et al. [30] observed that rs16944 SNP may be linked with HO in Saudi population. However, likewise, our findings were also quite different from theirs. They found percentage of genotype GG was lower while AA was higher in patient group than those in controls, and therefore, they concluded AA genotype may be a risk factor of HO. In our study, percentage of AA genotype was lower while AG genotype was higher in patient group, suggesting that AG genotype may a risk factor of COM. With regard to underlying mechanisms of rs16944 polymorphism in the development of COM, we compared preoperative serum levels of different inflammatory biomarkers. Results only showed that serum IL-6 level in AG genotype group was significantly higher than those in AA group and AA+GG group, implying that IL-6 may be one of the mediators in the development of IL-1*β* induced COM.

In this study, we also observed a trend that rs1143627 polymorphism may be linked with increased susceptibility to COM in Chinese Han population, though outcomes of dominant (*P* = 0.061), homozygous (*P* = 0.080), and heterozygous (*P* = 0.095) models were not statistically significant. It was also interesting that serum IL-6 level in CT genotype group was significantly higher than that in CC genotype group. Moreover, serum IL-6 level of CT+TT genotypes group was also statistically higher than CC group. Therefore, on the one hand, a larger sample size is quite necessary. On the other hand, further explorations are required to better understand potential role of IL-6 in the pathogenesis of rs16944 and rs1143627 polymorphisms induced COM.

The present study had several limitations. Firstly, although the sample size of the present study was larger than most of the published studies, it is still far from enough for SNP investigation. In addition, as mentioned previously, pathogenic mechanism for establishment of COM is multifactorial; therefore, larger number of participants should be included in future studies. Secondly, we did not detect serum IL-1 level of each case and therefore, association between IL-1*β* gene polymorphisms and serum IL-1 levels remains unclear. Thirdly, as mentioned previously, in addition to host genetic factors, environmental factors also involve in the pathogenesis of COM. Due to the fact that most of the COM patients included in our study were transferred from local hospitals, detailed medical information (e.g., severity of initial injury, treatment strategy, etc.) before bone infection was unclear. Therefore, we cannot perform further analysis regarding the role of such external factors in the development of COM. Fourthly, this study was a preliminary report. Further studies should be conducted for detailed mechanisms underlying this phenomenon.

In summary, the present study found that IL-1*β* gene rs16944 polymorphism may be correlated with increased risk of developing COM in Chinese Han population. People with genotype of AG may suffer from a higher susceptibility to COM.

## Figures and Tables

**Table 1:** Forward, reverse, and extension primers of the 4 tag SNPs for PCR.

SNPs	Forward and reverse primers (5′-3′)	Extension primers (5′-3′)
rs16944	F: ATGTGGGACAAAGTGGAAGAC	TTTTTTTTTTTTCTTGGGTGCTGTTCTCTGCCTC
	R: AATTTTCTCCTCAGAGGCTC	
rs1143627	F: CCACCAATACTCTTTTCCCC	TTTTTTTTTTTTTTTTTCCTACTTCTGCTTTTGAAAGC
	R: CCTTAGCACCTAGTTGTAAG	
rs1143634	F: CTACTGGTGTTGTCATCAGAC	TTTTTTTTTTTTTTTTTTAAGCCTCGTTATCCCATGTGTC
	R: AGCTTTTTTGCTGTGAGTCCC	
rs2853550	F: ACTAAAGCCCACTCCTCATTG	TTTTTTTTTTTTTTTTTTTTTCAGAAGGATATTCAGTGCACAT
	R:ATAGCGCTTGCTCAACAGATG	

SNPs: single nucleotide polymorphisms.

PCR: polymerase chain reaction.

**Table 2:** Comparisons of genotype distribution, allele frequency, and genetic models between COM patients and healthy controls.

SNP ID	Items		Patients	Healthy controls	*P* value	OR (95%CI)
rs16944	Genotype (n, %)	AA	40 (17.17)	52 (26.0)	0.079	NA
		AG	120 (51.50)	90 (45.0)		
		GG	73 (31.33)	58 (29.0)		
	Allele	G vs. A	266/200	206/194	0.100	1.253 (0.958-1.638)
	Dominant	GG+AG vs. AA	193/40	148/52	**0.026**	1.698 (1.065-2.707)
	Recessive	GG vs. AA+AG	73/160	58/142	0.586	1.122 (0.742-1.696)
	Homozygous	GG vs. AA	73/40	58/52	0.071	1.643 (0.958-2.818)
	Heterozygous	AG vs. AA	120/40	90/52	**0.030**	1.733 (1.055-2.847)
rs1143627	Genotype (n, %)	CC	42 (18.03)	51 (25.5)	0.163	NA
		CT	118 (50.64)	94 (47.0)		
		TT	73 (31.33)	55 (27.5)		
	Allele	T vs. C	264/202	204/196	0.096	1.256 (0.960-1.642)
	Dominant	TT+CT vs. CC	191/42	149/51	0.061	1.557 (0.980-2.473)
	Recessive	TT vs. CC+CT	73/160	55/145	0.368	1.211 (0.798-1.838)
	Homozygous	TT vs. CC	73/42	55/51	0.080	1.620 (0.944-2.779)
	Heterozygous	CT vs. CC	118/42	94/51	0.095	1.520 (0.930-2.485)
rs1143634	Genotype (n, %)	CC	229 (98.28)	194 (97.0)	0.524	NA
		CT	4 (1.72)	6 (3.0)		
		TT	0 (0.0)	0 (0.0)		
	Allele	T vs. C	4/462	6/394	0.574	0.569 (0.159-2.029)
	Dominant	TT+CT vs. CC	4/229	6/194	0.402	0.577 (0.160-2.084)
	Recessive	TT vs. CC+CT	0/233	0/200	NA	NA
	Homozygous	TT vs. CC	0/229	0/194	NA	NA
	Heterozygous	CT vs. CC	4/229	6/194	0.402	0.577 (0.160-2.084)
rs2853550	Genotype (n, %)	CC	206 (88.41)	171 (85.5)	0.281	NA
		CT	27 (11.59)	27 (13.5)		
		TT	0 (0.0)	2 (1.00)		
	Allele	T vs. C	27/439	31/369	0.251	0.732 (0.429-1.249)
	Dominant	TT+CT vs. CC	27/206	29/171	0.315	0.749 (0.425-1.317)
	Recessive	TT vs. CC+CT	0/233	2/198	0.999	0.000 (0.000-* *-* *-
	Homozygous	TT vs. CC	0/206	2/171	0.999	0.000 (0.000-* *-* *-
	Heterozygous	CT vs. CC	27/206	27/171	0.459	0.805 (0.454-1.429)

SNP: single-nucleotide polymorphism; OR: odds ratio; CI: confidence interval. NA: not available.

**Table 3:** Comparisons of clinical features among different genotypes of the IL-1*β* gene polymorphisms in COM patients.

Items	rs16944				rs1143627				rs1143634			rs2853550		
	AA	AG	GG	*P* value	CC	CT	TT	*P* value	CC	CT	*P* value	CC	CT	*P* value
Age Median (IQR)	42.50 (27.00, 50.00)	37.50 (24.00, 52.00)	39.00 (28.50, 48.50)	0.696	42.00 (27.00, 50.00)	37.50 (24.00, 52.25)	39.00 (28.50, 48.50)	0.838	39.00 (26.00, 50.00)	48.50 (39.50, 59.75)	0.182	39.00 (26.00, 50.25)	38.00 (27.00, 49.00)	0.769
Sex ratio (M/F)	28/12	100/20	57/16	0.185	30/12	98/20	57/16	0.263	182/47	3/1	1.000	163/43	22/5	0.776
Positive rate of culture (%) (E/T)	68.97 (20/29)	65.17 (58/89)	61.82 (34/55)	0.803	67.74 (21/31)	65.52 (57/87)	61.82 (34/55)	0.839	64.91 (111/171)	50 (1/2)	1.000	62.18 (97/156)	88.24 (15/17)	0.033
Polymicrobial infection (%) (E/T)	30.00 (6/20)	18.97 (11/58)	20.59 (7/34)	0.597	28.57 (6/21)	19.30 (11/57)	20.59 (7/34)	0.682	21.62 (24/111)	0 (0/1)	1.000	21.65 (21/97)	20 (3/15)	1.000
WBC (×10^9^/L) Median (IQR)	7.66 (6.33, 9.07)	7.34 (5.59, 9.36)	6.93 (6.11, 8.51)	0.414	7.66 (6.38, 9.16)	7.34 (5.55, 9.28)	6.93 (6.11, 8.51)	0.346	7.24 (5.94, 8.93)	7.30 (5.46, 7.90)	0.660	7.25 (6.03, 8.77)	7.39 (5.41, 9.99)	0.833
CRP (mg/L) Median (IQR)	3.99 (1.67, 33.36)	6.20 (2.28, 21)	7.65 (2.41, 18.58)	0.646	3.99 (1.48, 29.61)	6.20 (2.32, 21.20)	7.65 (2.41, 18.58)	0.567	6.25 (2.24, 19.70)	6.51 (2.31, 14.18)	0.844	6.57 (2.37, 19.35)	4.67 (1.57, 17.56)	0.459
PCT (ng/ml) Median (IQR)	0.037 (0.020, 0.053)	0.035 (0.023, 0.069)	0.038 (0.026, 0.051)	0.894	0.038 (0.020, 0.053)	0.035 (0.023, 0.070)	0.038 (0.026, 0.051)	0.912	0.037 (0.023, 0.062)	0.030 (0.024, 0.038)	0.499	0.037 (0.024, 0.062)	0.027 (0.020, 0.051)	0.273
IL-6 (pg/ml) Median (IQR)	7.98 (3.73, 18.17)	21.10 (8.71, 74.49)	12.44 (4.34, 53.46)	**0.005**	7.98 (3.60, 20.08	21.1 (8.84, 74.50)	12.44 (4.34, 53.46)	**0.003**	15.39 (6.55, 59.78)	24.29 (4.05, 38.95)	0.781	14.76 (6.22, 59.35)	24.67 (8.10, 77.43)	0.379
TNF-*α* (pg/ml) Median (IQR)	9.67 (7.67, 11.20)	9.78 (7.56, 13.05)	8.73 (7.21, 11.53)	0.494	9.7 (8.04, 11.60)	9.42 (7.55, 13.10)	8.73 (7.21, 11.53)	0.510	9.26 (7.38, 12.00)	9.04 (8.07, 12.77)	0.872	9.16 (7.50, 12.00)	10.40 (6.65, 13.50)	0.698
SAA (mg/L) Median (IQR)	7.40 (2.45, 28.25)	9.40 (4.20, 37.40)	5.00 (3.23, 61.75)	0.776	8.15 (2.48, 26.58)	8.55 (4.18, 37.53)	5.00 (3.23, 61.75)	0.861	7.60 (3.30, 29.05)	272.30	0.135	8.30 (3.33, 30.33)	5.25 (2.85, 222.05)	0.858

COM: chronic osteomyelitis; IQR: interquartile range; M/F: male/female; E/T: events/total; WBC: white blood cell count; CRP: C-reactive protein; PCT: procalcitonin; IL-6: interleukin-6; TNF-a: tumor necrosis factor-a; SAA: serum amyloid A.

## Data Availability

The datasets generated and/or analyzed during the current study are not publicly available due to the respect and protection of privacy of the patients but are available from the corresponding author upon reasonable request.

## Abbreviations

IL-1*β*: Interleukin-1 beta
SNPs: Single nucleotide polymorphisms
COM: Chronic osteomyelitis
VDR: Vitamin D receptor
COX-2: Cyclooxygenase-2
tPA: Tissue plasminogen activator
MMP-1: Matrix metalloprotease 1
EDTA: Ethylene diamine tetraacetic acid
WBC: White blood cell count
CRP: C-reactive protein
PCT: Procalcitonin
IL-6: Interleukin-6
TNF-*α*: Tumor necrosis factor-*α*
SAA: Serum amyloid A
SPSS: Statistical Product and Service Solutions
IQR: Interquartile range
ANOVA: One-way analysis of variance
HWE: Hardy-Weinberg equilibrium
OR: Odds ratio
CI: Confidence interval
VTE: Venous thrombosis
ESRD: End-stage renal disease.

## Additional Points

Interleukin-1 beta gene polymorphism rs16944 may associate with increased susceptibility to extremity chronic osteomyelitis in Chinese Han population.

## References

[1] Winkler H. (2017). Treatment of chronic orthopaedic infection. *EFORT Open Reviews*.

[2] Jiang N., Ma Y., Jiang Y. (2015). Clinical characteristics and treatment of extremity chronic osteomyelitis in southern china: a retrospective analysis of 394 consecutive patients. *Medicine (Baltimore)*.

[3] Tseng C. H., Huang W. S., Muo C. H. (2014). Increased depression risk among patients with chronic osteomyelitis. *Journal of Psychosomatic Research*.

[4] Ho M. W., Tseng C. H., Chen J. H. (2015). Chronic osteomyelitis as a risk factor for development of rheumatoid arthritis: a nationwide, population-based, cohort study. *Clinical Rheumatology*.

[5] Lai S. W., Lai H. C., Lin C. L., Liao K., Tseng C. (2015). Chronic osteomyelitis correlates with increased risk of acute pancreatitis in a case–control study in Taiwan. *European Journal of Internal Medicine*.

[6] Lin S. Y., Lin C. L., Tseng C. H. (2014). The association between chronic osteomyelitis and increased risk of diabetes mellitus: a population-based cohort study. *European Journal of Clinical Microbiology & Infectious Diseases*.

[7] Tseng C. H., Huang W. S., Muo C. H., Chang Y., Sung F. (2015). Increased risk of intracerebral hemorrhage among patients with chronic osteomyelitis. *Journal of Neurosurgery*.

[8] Metsemakers W. J., Smeets B., Nijs S., Hoekstra H. (2017). Infection after fracture fixation of the tibia: Analysis of healthcare utilization and related costs. *Injury*.

[9] Jiang N., Zhao X. Q., Qin C. H. (2016). Association of vitamin D receptor gene TaqI, BsmI, FokI and ApaI polymorphisms and susceptibility to extremity chronic osteomyelitis in Chinese population. *Injury*.

[10] Wang L., Jiang N., Lin Q. R., Qin C., Hu Y., Yu B. (2017). Cyclooxygenase-2 (COX-2) polymorphism rs689466 may contribute to the increased susceptibility to post-traumatic osteomyelitis in Chinese population. *Infectious Diseases*.

[11] Valle-Garay E., Montes A. H., Corte J. R., Meana A., Fierer J., Asensi V. (2013). tPA Alu (I/D) polymorphism associates with bacterial osteomyelitis. *The Journal of Infectious Diseases*.

[12] Montes A. H., Valle-Garay E., Alvarez V. (2010). A functional polymorphism in MMP1 could influence osteomyelitis development. *Journal of Bone and Mineral Research*.

[13] Lukens J. R., Gross J. M., Calabrese C. (2014). Critical role for inflammasome-independent IL-1 production in osteomyelitis. *Proceedings of the National Acadamy of Sciences of the United States of America*.

[14] Gurung P., Burton A., Kanneganti T. D. (2016). NLRP3 inflammasome plays a redundant role with caspase 8 to promote IL-1*β*–mediated osteomyelitis. *Proceedings of the National Acadamy of Sciences of the United States of America*.

[15] Chen S. T., Ni Y. H., Liu S. H. (2018). Potential association of IL1B polymorphism with iron deficiency risk in childhood helicobacter pylori infection. *Journal of Pediatric Gastroenterology and Nutrition*.

[16] Kasztelewicz B., Czech-Kowalska J., Lipka B. (2017). Cytokine gene polymorphism associations with congenital cytomegalovirus infection and sensorineural hearing loss. *European Journal of Clinical Microbiology & Infectious Diseases*.

[17] Jimenez-Sousa M. A., Medrano L. M., Liu P. (2017). IL-1B rs16944 polymorphism is related to septic shock and death. *European Journal of Clinical Investigation*.

[18] Rogo L. D., Rezaei F., Marashi S. M. (2016). Seasonal influenza A/H3N2 virus infection and IL-1Β, IL-10, IL-17, and IL-28 polymorphisms in Iranian population. *Journal of Medical Virology*.

[19] Panteli M., Giannoudis P. V. (2016). Chronic osteomyelitis: what the surgeon needs to know. *EFORT Open Reviews*.

[20] Huang C. C., Tsai K. T., Weng S. F. (2016). Chronic osteomyelitis increases long-term mortality risk in the elderly: a nationwide population-based cohort study. *BMC Geriatrics*.

[21] Lin T. Y., Chen Y. G., Huang W. Y. (2014). Association between chronic osteomyelitis and deep-vein thrombosis Analysis of a nationwide population-based registry. *Thrombosis and Haemostasis*.

[22] Lin S. Y., Lin C. L., Tseng C. H. (2015). Association between chronic osteomyelitis and risk of end-stage renal disease a nationwide population-based cohort study. *Medicine*.

[23] Tseng C., Huang W., Muo C., Kao C. (2015). Increased risk of dementia among chronic osteomyelitis patients. *European Journal of Clinical Microbiology & Infectious Diseases*.

[24] Wang H., Chao C., Lin C., Tseng C., Kao C. (2016). Increased subsequent risk of erectile dysfunction among middle and old age males with chronic osteomyelitis: a nationwide population-based cohort study. *International Journal of Impotence Research*.

[25] Panteli M., Puttaswamaiah R., Lowenberg D. W., Giannoudis P. V. (2014). Malignant transformation in chronic osteomyelitis: recognition and principles of management. *Journal of the American Academy of Orthopaedic Surgeons*.

[26] Marais L. C., Ferreira N., Aldous C., Sartorius B., Le Roux T. (2015). A modified staging system for chronic osteomyelitis. *Journal of Orthopaedics*.

[27] Asensi V., Alvarez V., Valle E. (2003). IL-1*α* (− 889) promoter polymorphism is a risk factor for osteomyelitis. *American Journal of Medical Genetics Part A*.

[28] Tsezou A., Poultsides L., Kostopoulou F. (2008). Influence of interleukin 1 (IL-1 ), IL-4, and IL-6 polymorphisms on genetic susceptibility to chronic osteomyelitis. *Clinical and Vaccine Immunology*.

[29] Alves De Souza C., Queiroz Alves De Souza A., Queiroz Alves De Souza M. d., Dias Leite J. A., Silva De Morais M., Barem Rabenhorst S. H. (2017). A link between osteomyelitis and IL1RN and IL1B polymorphisms-a study in patients from Northeast Brazil. *Acta Orthopaedica*.

[30] Osman A., Mubasher M., ElSheikh N. (2016). Association of single nucleotide polymorphisms in pro-inflammatory cytokine and toll-like receptor genes with pediatric hematogenous osteomyelitis. *Genetics and Molecular Research*.

